# Restoration success in former Amazonian mines is driven by soil amendment and forest proximity

**DOI:** 10.1098/rstb.2021.0086

**Published:** 2023-01-02

**Authors:** Louis A. König, José A. Medina-Vega, Regina M. Longo, Pieter A. Zuidema, Catarina C. Jakovac

**Affiliations:** ^1^ Wageningen Environmental Research (WENR), Wageningen University and Research, Droevendaalsesteeg 3, 6708 PB, Wageningen, The Netherlands; ^2^ Forest Ecology and Management Group, Wageningen University, Droevendaalsesteeg 3, 6708 PB, Wageningen, The Netherlands; ^3^ Forest Global Earth Observatory, Smithsonian Tropical Research Institute, Washington, DC 20013, USA; ^4^ Pontifical Catholic University of Campinas, Professor Doutor Euryclides de Jesus Zerbini, 1516, 13087-571 Campinas, Brazil; ^5^ Plant Sciences Department, Center for Agrarian Sciences, Federal University of Santa Catarina, Rodovia Admar Gonzaga, 1346, 88040-900, Florianópolis, Brazil

**Keywords:** ecological drivers, management, mine land restoration, natural regeneration, planting, tropical forest

## Abstract

Mining contributes importantly to tropical deforestation and land degradation. To mitigate these effects, mining companies are increasingly obliged to restore abandoned mine lands, but factors driving restoration success are hardly evaluated. Here, we investigate the influence of ecological factors (restoration age, soil properties and surrounding forest area) and management factors (diversity and density of planted species, mine zone) on the recovery rate of forest structure and tree diversity on 40 post-mining restoration areas in Southern Amazonia, Brazil, using a 9-year annual monitoring dataset consisting of over 25 000 trees. We found that recovery of forest structure was closely associated with interactions between soil quality and the planted tree communities, and that tree diversity recovery was positively associated with the amount of surrounding forests. We also observed that forest structure and diversity recover more slowly in mine tailings compared to pit surroundings. Our study confirms the complexity of mine land restoration but also reveals that planting design and soil improvement can increase restoration success. For resource-efficient mine restoration, we recommend the focusing of efforts on tailings, which are hardest to restore, and reducing efforts in pit surroundings and areas close to surrounding forest because of their potential for restoration by natural regeneration.

This article is part of the theme issue ‘Understanding forest landscape restoration: reinforcing scientific foundations for the UN Decade on Ecosystem Restoration’.

## 1. Introduction

The restoration of degraded forest areas in the tropics has lagged behind that of temperate and boreal zones, but is gaining momentum now [1]. This is reflected by the substantial pledges made by tropical countries to restore degraded forest land as part of the Bonn Challenge and New York Declaration on Forests. These ambitious restoration commitments are supported by a recent overview of methods [2], comparison of approaches [3] and identification of suitable locations [4] for tropical forest restoration. Yet, most restoration activities are still implemented in a trial-and-error fashion and systematic analyses of the factors determining restoration success are scarce [5]. As a result, advice on which restoration approaches to use in what situations is often formulated in general terms [6]. Analyses of success are crucial to develop practical guidelines for effective restoration but require sound documentation of restoration measures and intensive monitoring [7]. A central challenge in assessing restoration success is to quantify the relative roles of ecology versus active management [8]. Management factors relate to the soil treatment applied, the diversity and composition of planted tree species and the temporary use of exotics to kickstart regeneration [9]. Ecological factors include the availability of seed sources from surrounding forests, natural successional processes and soil characteristics [10]. The relative importance of these factors is a central point of discussion in ecological restoration literature [11], but quantitative analyses of their roles are missing so far.

One of the severest forms of degradation in tropical forests is open pit mining. Mining contributes importantly to total tropical deforestation (approx. 9% in the Amazon) [12], but mining companies are stimulated or legally obliged to restore forests on former mine lands towards self-sustaining ecosystems [13]. As a result, vast areas of abandoned surface mines are now actively restored [14,15]. The restoration of open pit mines constitutes a challenge because of topsoil removal and soil deposition in tailings [16]. Mine restoration is therefore an intensive endeavour and often requires soil amendment and topographic modification [17], followed by active tree planting [10] and natural regeneration [18]. Together, the demanding mine restoration process and the vast extent of abandoned mines form a formidable challenge for ecological restoration. Analyses of the factors determining success of past mine restoration processes are therefore urgently needed.

**Figure 1.**
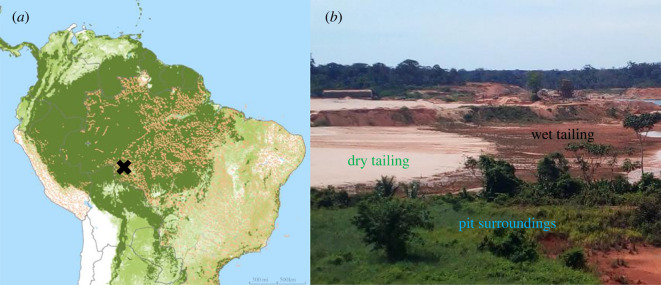
(*a*) Study location (black cross) in the Brazilian Amazon region together with tree cover (light green), primary forest extent (dark green) and active mining concessions (orange; global forest watch). (*b*) Picture of one of the studied open-pit mines in Brazil, indicating three mine zones. Capped tailings—tailings on which topsoil is transplanted—are not included here; washed tailings are not included as they result from a different mining technique. (Online version in colour.)

A specific challenge in mine restoration is to adapt to the strong heterogeneity in soil conditions created by mining operations [19]. The main mine zones created during operations are the surroundings of the pit, where the original soil layering is preserved but might be compacted, and the tailing dam, where the soil is unstructured as a result of washing and mixing (figure 1*b*) [20]. Current research typically investigates restoration success on specific tailing damn types, neglecting the presence and heterogeneity of other mine zones, but recognizes that restoration techniques should take into account differences in microhabitats [21,22].

This study aimed to determine the influence of ecological and management factors on the success of mine land restoration and to interpret their implications for restoration practices. We monitored more than 25 thousand trees in 40 restoration areas (aged 10–25 years) that were located in 7 deactivated mines in the Amazon basin (Rondônia, Brazil) over a 9‐year period. We quantified restoration success as the recovery of forest structure (basal area, stem density) and diversity (species richness and diversity) over time. We tested contributions of ecological (soil physical and chemical properties, restoration age and landscape context) and management factors (mine zone, planted stem density, planted species richness and planted species composition) to recovery. We hypothesize that (i) the recovery of forest structure is mainly driven by management factors, while (ii) that of forest diversity is governed by both management and ecological factors. In all analyses, we also tested for effects of mine zones on recovery. Our systematic assessment of driving factors for mine restoration success contributes to the development of tailored and resource-efficient restoration guidelines. Expanding the knowledge base on how disturbances affect soil conditions and therefore restoration success as well as on the impacts of using native and exotic species in restoration is considered crucial for achieving global forest restoration targets [23].

## 2. Material and methods

### (a) Study location

We conducted this study in the Jamari National Forest, located in the municipality of Itapuã do Oeste, Rondônia state, Brazil; within the Amazon biome (figure 1*a*).

The Jamari National Forest spans 220 000 ha, in which mining and logging are allowed. Data were collected in seven deactivated Casserite mines (Casserite is a mineral used for tin production). Together with one other active mine, the mining area accounts for 10% of the national forest. The other 90% is covered by evergreen tropical forests. Climate is tropical wet with a well-defined dry season (less than 100 mm monthly precipitation) from May to September and wet season from October to April (monthly precipitation from 200 to 400 mm). Mean annual temperature is 22°C and mean annual precipitation is 2500 mm. The dominant soil types in the study area are Oxisols, Ultisols and hydromorphic soils.

### (b) Mine zones

Two mining techniques were applied: pit mining and washing, which involve different levels of substrate modification that were grouped into mine zones for the restoration action [19]. In pit mining, pit holes are dug and sterile soil material is deposited on tailing dams, resulting in four mine zones: pit surroundings, wet tailing, dry tailing and capped tailing (figure 1*b*). Pit surroundings were used for transportation, and operating heavy mining machinery, leading to physical soil compaction but keeping the original soil structure*.* Mine tailings are dams of soil deposits composed of unstructured sterile soils with a slope that allows for drainage. The combination of slope and long-term exposure creates a gradient of soil particle sizes, with sandy soils on the top and clayey soils on the bottom. Three mine zones are differentiated along the slope of the dams. First, ‘dry tailings' are the upper dam zones and characterized by higher sand content that causes dry soil conditions through lower water retention capacity and a loose soil structure due to a lack of heterogeneity in soil particle sizes. Second, ‘wet tailings' are the lower portions of the dam, characterized by the dominance of finer soil particles (i.e. clay and silt). These areas can become waterlogged due to high clay content and their position in the landscape. Third, ‘capped tailings' are present in some cases when the dry or wet tailings are covered with topsoil obtained from recent forest openings elsewhere, and thus richer in organic matter, soil biota and seeds.

In mines where washing is used, no pit hole is dug and the mineral is extracted by mobile machinery that removes shallow soil layers, extracts the mineral and deposits the washed sterile soil back in its original location. Washing results in one mining zone, ‘washed tailings'. The soil texture in washed tailings is similar to original soil and thus differs from wet, dry and capped tailings. Washed tailings are not exposed to slopes, preventing the separation of soil particles and resulting in a heterogeneous soil structure [24].

### (c) Restoration activities

Before trees were planted, soil quality, organic matter content and soil biology were improved for three years using a combination of chemical fertilization, liming, cow manure and green manure. Then, nine restoration areas (all capped tailings) were selected for topsoil transplantation based on the nutrient availability determined by soil analyses. In all areas, chemical fertilization was applied during tree planting and continued for at least 5 years to provide macro-nutrients (N, P, K) for successful sapling establishment.

Because no rapid natural tree regeneration took place in the areas, active tree planting was required. Tree saplings were grown in a local tree nursery from seeds obtained from easily accessible seed trees located inside and outside the national forest. Planting densities and species mixture varied among sites. Reforestation started in 1991 and activities are still ongoing (December 2021); the mean start year of reforestation was 2003 (s.d. ± 2.7 years). A total of 105 tree species were included in planting (electronic supplementary material, S1) and on average 1680 (s.d. ± 1059) trees were planted per hectare. The two dominant planted tree species were *Syzigium cumini* (Myrtaceae), an exotic species originating from the Indian subcontinent, and the native *Inga laurina* (Fabaceae)*,* a fast-growing legume. These species were planted in high abundance, particularly at the beginning of the restoration activities, because they withstand harsh environmental conditions such as low nutrient content, degraded soil biota, low water availability in the dry tailings or water logging in the wet tailings. The exotic species were used to colonize the sites to initiate the establishment of natural regeneration. The exotic trees were removed after fulfilling this function.

### (d) Data collection

We recorded each individual's taxonomic identity and size in 89 permanent plots located in 40 restoration areas in seven deactivated mines (electronic supplementary material, S2) annually from 2009 to 2017. Each plot was 50 × 50 m and contained six subplots of varying dimensions where different tree and seedling sizes were sampled (see electronic supplement material, S3 for inclusion criteria). The size of the studied mines ranged from 6 to 196 ha. The mines were divided into restoration areas depending on their former location in the mine. Their sizes ranged from 0.4 to 135.6 ha, and age since planting ranged from 10 to 26 years (in 2017).

Annual monitoring was conducted during the dry season. Diameter at breast height (DBH) was taken for trees with a DBH > 10 cm. All trees and saplings taller than 0.3 m were identified to the highest taxonomic level possible by local parataxonomists. Additionally, we identified whether each individual tree was regenerated naturally or planted. Similar measurements were conducted in 26 reference plots (in 2010) in secondary forests outside the mines (aged 15–30 years) and in 24 reference plots in old-growth forests surrounding each mine.

#### (i) Composition of regeneration method and tree species origin

We calculated the proportion of planted and naturally regenerated trees for the beginning (2009) and the end (2017) of the study period using the tree observations from all restoration areas. We then determined the proportion of exotic and native trees for the classes 'planted' and 'naturally regenerated' trees.

#### (ii) Soil variables and landscape configuration

**Figure 2.**
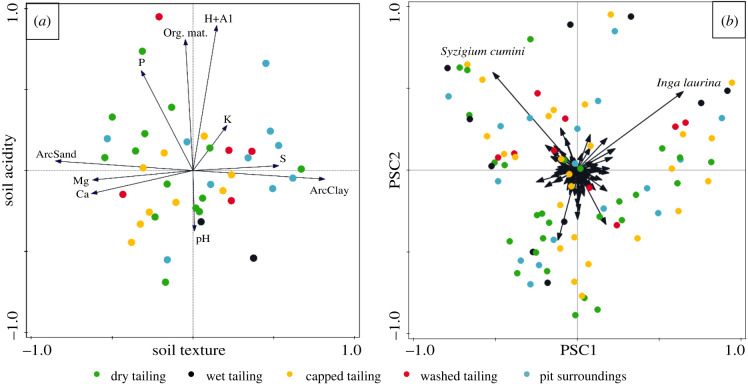
(*a*) PCA-biplot of soil samples (dots represent monitoring plots, colour-coded by mine zone) and the measured variables (arrows). Sand and clay content were arcsine transformed (referred to as ArcSand and ArcClay). The first two components explain 45.7% of the total variation (26.7% by first component). (*b*) PCoA-biplot of the planted species composition (dots, colour-coded by mine zone) and the planted species (arrows). The first two components explain 22.9% of the variation (12.85% by first component). PSC stands for planted species composition. (Online version in colour.)

The composition of sand, silt and clay was measured in 2011. We collected three soil samples per restoration area and measured pH, organic matter, sulfate, calcium, magnesium, potassium, aluminium and total soil phosphorus (using the Mehlich-1 method) in 2017 following Raij *et al*. [25]. The mean value per restoration area was used in further analyses. Soil particle fractions were analysed on soil samples of 1000 g. We constructed a principal component analysis (PCA; figure 2) including all soil variables and used the calculated scores of the first two components to describe the soil properties in subsequent analyses.

We characterized the landscape context for each plot using seven proxies: the area of surrounding forest in buffer circles with radii of 500, 300, 200, 100 m, the distances to secondary and old-growth forest, and the size of the restoration area. These proxies were strongly correlated (*r* = 0.2 to 0.91; Pearson) and we therefore selected the best landscape proxy by comparing a set of (preliminary) linear mixed effect models. Each model included one of the seven landscape variables as covariate. The best landscape proxy (i.e. the model with lowest AIC) for the relationship between landscape and biodiversity recovery was the area of surrounding forest (secondary and old-growth forest) within a radius of 500 m.

### (e) Statistical analyses

#### (i) Rationale and preparation of response variables

In active restoration, trees are planted to create a favourable micro-environment that allows native species to regenerate, and succession to proceed [26]. We investigated the development in forest structure including both planted and naturally regenerating trees and evaluated changes in forest diversity including only naturally regenerated trees. For forest structure, we calculated plot basal area and stem density as the sum of the planted and naturally regenerating trees from all subplots after extrapolating to a hectare. For species richness, we summed the total number of naturally regenerating species per plot. Since there were often only few species present within monitoring plots at early stages of succession (*ca* 1.5 species per plot in 2009), rarefaction was not applied because it would result in a very low maximum species richness. For species diversity, we calculated the Shannon diversity index including only naturally regenerating trees after extrapolating the species abundance data of the subplots to one hectare.

We expressed restoration success as the increment rate of basal area and stem density for forest structure recovery, and as the increment rate of species richness and species diversity for species diversity recovery. We estimated recovery rates of basal area, density, species richness and diversity using linear mixed effects models with normally distributed errors, calendar year (i.e. multiple census) as main explanatory variable, and plot ID and calendar year as random intercept and random slope, respectively. These models yielded estimates of the rates of recovery at the community level via fixed effects and at the plot level via random effects (i.e. random slopes). This random structure also allowed us to control for multiple observations per plot. Plot-level coefficients served as our estimates for the increment rates per plot, a measure of restoration success, and used in subsequent analyses. We additionally included area ID nested within mine ID as additional random intercepts to control for the nested and unbalanced structure of the design and the higher relatedness among closely located plots. Year was centred and standardized prior to the analyses.

#### (ii) Covariate's specification and multiple regression models

We related the recovery rates of basal area, stem density, species richness and diversity to five management variables (i.e. planted species richness, planted tree density, two principal component analyses (PCoA) axes of planted species composition (electronic supplementary material, S4) and mine zones), four ecological variables (i.e. age of the restoration areas (centred at the midpoint of the monitoring period: 2013), two soil PCA axes (electronic supplementary material, S4), and the amount of area of surrounding forest within 500 m distance) and 13 interactions using linear models with normally distributed errors (electronic supplementary material, S5). For tree density recovery rates, we used a (generalized) linear model with Gamma-distributed errors. All continuous variables were centred and standardized. For each response variable, we first compared models that included fixed and random terms (areas nested within mines) with models that included only the fixed terms by using AIC and the restricted maximum-likelihood estimator. Models without random terms had lower AIC values. We then simplified the models by removing interaction terms and variables that did not improve the predictive quality of the models. The best-fit model for each analysis was selected using AIC [27]. We considered models with DAIC of less than 4 to have a similar level of empirical support from the data and selected the most parsimonious model when two models had similar AIC values. Descriptive statistics of response and explanatory variables are documented in electronic supplementary material, S6.

We constructed all models using the lme4 package v. 1.1-23 [28] in R v. 4.0.0 [29] to estimate recovery rates and the first set of mixed models (fixed versus fixed + random terms). We used the base R function ‘lm' for basal area, species richness and species diversity and the function ‘glm’ for stem density. Model validation was assessed graphically as detailed in Zuur & Ieno [30]. We finally estimated the precision of model terms by computing the 95% confidence interval of the parameter estimates using 1000 parametric bootstrap simulations and the percentile interval method.

## 3. Results

### (a) General restoration dynamics

The share of planted trees declined from almost 70% to 12% throughout the study period and, simultaneously, the proportion of planted exotic trees halved. The natural regeneration is dominated by the establishment of native trees. The share of naturally regenerated exotic trees declined from 9.8% to 2.0% (table 1).

**Table 1.** Percentages of different regeneration methods (planted and natural) and species origins (native and exotic) as well as their combinations for stem density at the beginning (2009) and the end (2017) of the monitoring period.

	year	regeneration method		planted trees		natural regeneration	
		planted	natural	native	exotic	native	exotic
stem density	2009	68.6	31.4	64.6	35.4	90.2	9.8
	2017	12.0	88.0	82.3	17.7	98.0	2.0

### (b) Soil properties

The first axis of the PCA on soil properties explained 26.7% of variation (figure 2*a*) and represents plots with high sand content, Mg and Ca in the negative side of the axis, and plots with high clay content, low magnesium and calcium in the positive side of the axis. We hereafter refer to this axis as ‘soil texture'. The second component on soil properties explained 19% of variation and revealed a gradient from plots with high to low pH (4.4–3.6), organic matter, potassium, phosphorus and aluminium. We hereafter refer to this axis as ‘soil acidity' (figure 2*a*). The PCA separated mine zones by physical soil properties, with dry and capped tailings associated with a high sand content, and wet tailings and pit surroundings with higher clay content. The areas on the washed tailings were distributed across the full range of the first axis, indicating a high variability in soil particle compositions in this mine zone, corresponding to the soil treatment in this mining type. The chemical soil properties were not clearly linked to mine zones.

### (c) Planted species composition

The first two PCoA axes of the planted species composition (figure 2*b*) explained 22.8% of the variation. The species *Syzigium cumini* and *Inga laurina* contributed most to the ordination along both axes due to their overall dominance in planted species composition, while the other more equally planted species among plots contributed less to the variation of the planted species composition. The first PCoA axis (hereafter PSC1) ordinates areas based on the dominance of *I. laurina* (positive values) or *S. cumini* (negative values). The second PCoA axis (hereafter PSC2) shows a gradient from low dominance of these species (negative values) towards high dominance by both species (positive values). Mine zones were not distinguished based on species composition.

### (d) Speed of forest recovery

**Figure 3.**
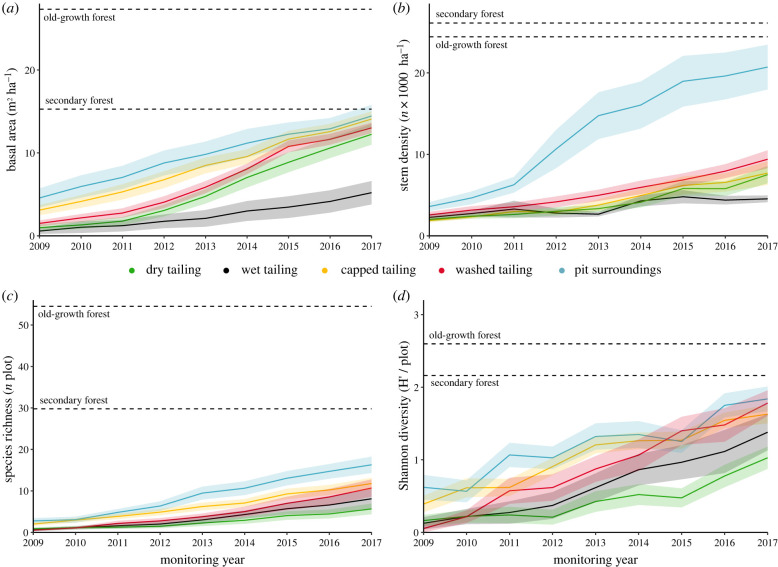
Recovery of forest structure and species diversity in restored open-pit mining areas in Brazil. The lines show the mean and the standard error in a given year and are colour-coded by mine zone. Dotted lines display average values for surveyed reference plots in secondary and old-growth forest in the immediate surroundings of the restoration areas. (Online version in colour.)

We observed a steady increase of forest structure and diversity over time (figure 3). Reference values from secondary forest for basal area and Shannon diversity index were reached by 30% and 20% of the restoration plots, respectively. Restoration sites showed a mean basal area recovery of 1.33 m^2^ y^−1^ and the mean basal area in 2017 (12.5 [s.d. ± 5.5] m^2^ ha^−1^) was close to the value of surrounding secondary forest (15.2 m^2^ ha^−1^) and half the value of nearby old-growth forests (27.3 m^2^ ha^−1^). Stem density recovery rates ranged between −112 and 6783 y^−1^ with a mean of 1001 new stems per year, indicating different successional stages across the sites. Mean stem density (10 000 stems ha^−1^) in 2017 recovered to roughly 40% of the secondary forest values (26 000 stems ha^−1^) and old-growth forests (24 000 stems ha^−1^) but variation across restoration sites was large (8740 stems ha^−1^) and some sites exceeded the stem numbers of old-growth forests (figure 3). Species richness showed a constant increase of 1.15 species per year with a standard deviation of 0.15, and species diversity recovery had the largest variability across restoration sites and increased on average by 0.15 per year (electronic supplementary material, S6).

### (e) Factors associated with forest structure recovery

**Figure 4.**
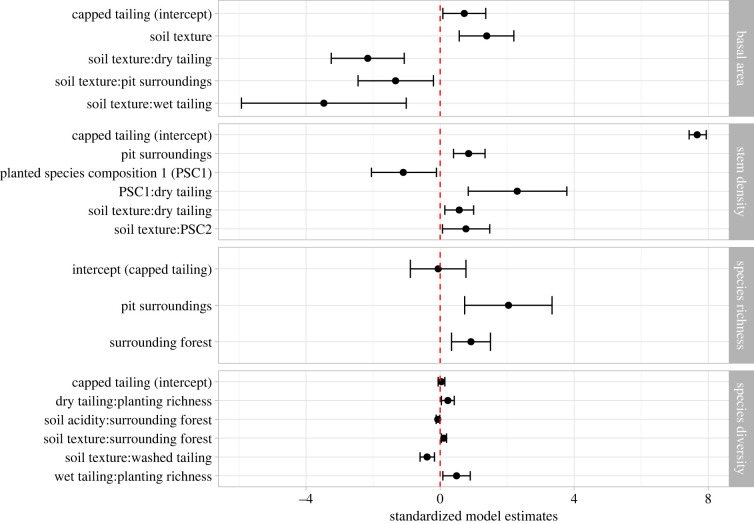
Significant effects of ecological and management factors (and interactions between them, shown by a colon) on the recovery of forest structure and forest diversity. The black dots show the standardized estimates of significant model parameters together with the associated confidence intervals. Points to the left of the dashed line show negative influence, while those on the right show positive influence. The full detailed model results are documented in electronic supplementary material, S7. Because of the inclusion of the categorical mine zone variable, capped tailings form the reference level of the model, denoted as intercept. (Online version in colour.)

The recovery rate of basal area was explained by the interaction between mine zones and soil texture (figure 4; electronic supplementary material, S7), accounting for 35% of the variation in recovery rates of basal area. Increasing clay content led to higher recovery rates of basal area in capped tailings but lower recovery rates in dry tailings.

The rate of stem density increment was related to both axes of the planted species composition, the mine zones and their interaction (figure 4). The model explained 49% of the variation in stem density increment across plots. Pit surroundings showed consistently higher rates of stem density increment compared to the dry and wet tailings and no difference from washed tailings. The interaction between mine zones and species composition indicates that in the dry and capped tailings (associated with sandy soils), density recovery rates were associated with species composition of the planted trees. Generally, in sandy soils, the abundance of *S. cumini* and *I. laurina* (PSC2) was associated with lower recovery rates of stem density. On dry tailings, however, stem density increment increased with clay content, with increasing densities of *I. laurina* and with decreasing densities of *S. cumini* (PSC1). The effect of these two species was the reverse in the capped mine tailings (figure 4).

### (f) Factors associated with forest diversity recovery

The increment of species richness increased with the area of surrounding forest and, similar to stem density increment, was higher in the pit surroundings compared to other mine zones (figure 4; electronic supplementary material, S7), explaining 28.6% of the variation.

Species diversity (Shannon index) was related to interactions between the area of surrounding forest, mine zones, planting parameters and soil texture. Constrained by soils with high pH and clay content, species diversity recovered faster with increasing amount of the surrounding forest. Higher planted species richness had a positive effect in the dry and wet tailings, suggesting that planted diversity is more crucial for restoration in these mine zones where there are more constraints to natural regeneration. In the washed tailings increasing clay content caused slower recovery rates.

## 4. Discussion

Overall, the results show that within 21 years of restoration, post-mining sites can attain values of vegetation structure and tree species diversity similar to secondary forests. The rates of recovery, however, varied enormously and were related to the level of soil transformation (mine zones and soil texture), the composition and richness of planted trees and the proximity to surrounding forests. Management practices of soil amendment and tree planting played a crucial role in the recovery of basal area, and combined with ecological processes related to seed dispersal and establishment of natural regeneration they determined the recovery of tree density and species diversity. These results show the complexity of forest restoration in post-mining areas and the need to define restoration practices tailored to the soil conditions and the landscape context.

We found that the recovery of forest structure was mainly affected by management practices (interactions between the planted species community, soil properties and mine zones), whereas the recovery of forest diversity was affected by the combination of management practices and ecological processes (amount of surrounding forest, soil texture and richness of planted trees). Similarly, in naturally regenerating forests, the recovery of vegetation structure is reduced where soil fertility is low and the soil seed bank had been impoverished, while the recovery of species richness and diversity is more related to the proximity to forest fragments [7]. The processes that govern forest recovery in active restoration are, therefore, similar to those in naturally regenerating forests [21,31,32].

Accordingly, we showed that forest structure recovers faster than species diversity (figure 3), as also found by other studies on natural regeneration [33] and active restoration [34]. Multiple areas reached basal area and stem density values similar to surrounding secondary forests within 12 years of restoration on average, while diversity took at least much longer (figure 3). This is because in highly diverse tropical forest systems, the recovery of diversity dependents on the recruitment of native species and its accumulation over time [2]. In active restoration of very degraded areas, such as in post-mining sites, tree planting contributes to the formation of a canopy that increases the density and diversity of natural regeneration [35,36]. Only in situations of extreme degradation, such as in the tailing dams, where natural regeneration is limited by constraints in soil conditions, did the recovery of diversity depend more strongly on the planted trees. Therefore, for a combined recovery of forest structure and diversity, it is crucial that areas are made permeable to the arrival and establishment of natural regeneration.

We showed that natural regeneration is denser and more diverse closer to the surrounding forests and on less degraded soil conditions. Previous studies on tropical successional forests also found higher species richness [31], species diversity [37] and higher density of late successional species [38] closer to old-growth forests. The positive effect of proximity to the forest, however, is only realized on less degraded soils. Interestingly, we found that natural regeneration contributed to a faster increase in stem density than in species diversity, likely due to the high abundance of a few generalist tree species that dominate the seed rain in regenerating forests [35,39]. Still, the influx of species led to an increased overlap of species composition between restoration areas and surrounding forests over time. Therefore, the natural regeneration under the canopy of the planted trees contributes importantly to the restoration success and can be enhanced by restoring the forest cover in the landscape and improving local soil conditions.

Interestingly, the species composition of planted trees had limited influence on restoration success, corroborating previous studies [40]. Despite the large number of planted species (105), *S. cumini* (exotic) and *I. laurina* (native) dominated the plantings and could potentially exert high influence on restoration success. These species were planted in high densities because they are tolerant to a wide range of soil conditions and were able to survive and grow in the harsh conditions where other species were not. For example, these two species are specially highly dominant in the dry tailings, where we also found low establishment rates of natural regeneration. It is unclear whether this low natural regeneration is caused by the poor soil conditions or by the competition for light and nutrients with the dominant species [41] or by the combination of both. This aspect could be further evaluated with population dynamics analyses. Nevertheless, in the other less-limiting substrates, we found no evidence relating the identity or origin (exotic or native) of these dominant species to the restoration success. Other studies found negative effects of planted exotics on the recovery of disturbed soils and on the establishment of native natural regeneration [42–44]. In this study, however, the exotic species showed low invasiveness potential, significantly decreasing in density over the years and showing low recruitment success (table 1). Although our study shows that this exotic species did not hinder restoration, we also show that it had similar behaviour to the native *I. laurina*, which should therefore be preferred in programs that aim to restore the native flora. These results suggest that the composition of tree plantings has limited effect on restoration success, that a high dominance by one species might be detrimental, and that a mine-zone specific selection of planted species [26] may improve restoration success. Further research is needed to disentangle the effects of planted species compositions and site conditions on restoration success [42].

In mined areas, the deep transformations in the soil structure form the most important factor negatively affecting restoration success. We found that in open-pit mines the chances of restoration success decreases with the intensity of soil transformation, being higher for pit surroundings and washing planting (where the original soil is maintained) and lower for wet and dry tailings (where the original soil has been lost). Pit surroundings and washed tailings provide more favourable conditions for natural regeneration and tree growth because they keep a structured soil profile and more active soil biota [45]. The 3-year soil amendment applied to all mine zones before tree planting proved efficient in improving soil quality and restoration success for most mine zones [24], but was not sufficient to correct soil physical characteristics of dry and wet tailings. In tailing dams, the transplantation of topsoil helped improve the recovery of forest structure, as seen by the higher recovery rates in capped compared to dry and wet tailings. We estimated that topsoil transplantation to tailings increases the rate of basal area growth by 13%, meaning a reduction of 3.7 years needed to achieve levels similar to the surrounding secondary forests. The transplanted topsoil introduces clay and silt particles that sustain soil water retention capacity, organic matter content, nutrient availability, soil biota and seed availability [14], supporting higher survival and growth rates of recruiting trees [41]. Our results thus confirm the importance of soil structure and the effectiveness of topsoil transplantation to improve soil conditions for tree growth and the establishment of natural regeneration.

In contrast to our predictions, restoration success was not significantly related to age. Explanations for this include: (i) the young age of restoration sites (less than 21 years old) when recovery shows a close-to-linear trend [32], (ii) the stronger influence of other factors on recovery rates (discussed below) and (iii) the improvement of restoration practices applied over time. According to the local restoration managers, past restoration practices (before 2003) included only the application of lime and chemical fertilizers followed by tree planting, while in more recent years (after 2003) soil was amended for 2 years using a combination of chemical fertilizers and green manure before tree planting. Additionally, the diversity of seedling species produced in the local tree nursery increased over time, allowing a higher diversity of species to be used in total-area plantings and in enrichment plantings. The absence of an age effect reinforces the potential of adaptive management practices to foster forest restoration success.

### (a) Implications and recommendations for post mining restoration

This study therefore shows how crucial the soil conditions and the proximity to forest fragments are for the success of restoration. Tailoring restoration strategies according to soil conditions and distance to seed sources (forest fragments) can increase the success of restoration and reduce costs. In tailing dams, not only do planted trees perform worse but also the natural regeneration cannot establish, even if the area is adjacent to a forest fragment. Tailing dams therefore represent the largest challenge for open mines' rehabilitation and restoration, requiring a large effort to restore soil structure prior to tree planting. Corrections to soil particle composition are necessary to restore the soil texture and hydrology of tailings and allow the survival and growth of a range of native species. Topsoil transplantation into tailings can help to foster restoration [14,40]. Ideally, other mine waste techniques that minimize the separation (or promote the mixture) of soil particles in tailing dams should be investigated as a way to increase the chances of restoration success [42,43,46].

Tree planting is crucial for the rehabilitation and restoration of mined areas because it facilitates the recruitment of native species and the re-establishment of the successional process [47]. In landscapes with high forest cover and therefore high availability of seeds, restoration projects should invest more in restoring soil chemical and physical conditions than in planting a high diversity of tree species.

## 5. Conclusion

This study confirms the complexity of mine land restoration but also the effectiveness of soil amendment and tree planting for restoration success. While forest structure can be restored based on management of the restoration areas, restoring forest diversity requires the assistance of surrounding seed sources and dispersers. To increase the restoration success in post-mining areas, it is crucial to continue evaluating the limitations imposed on succession, and zoning the area according to soil chemical and physical conditions and distance to the surrounding forest (i.e. in mine zones). Restoration plans should be tailored to each mine zone to increase restoration success and reduce implementation costs.

## Data Availability

The code and data to reproduce the multiple regression model results are provided in the electronic supplementary material: Code_multiple_regression_models.R and Data_multiple_regression_models.xlsx [48].
